# Genes Associated with SLE Are Targets of Recent Positive Selection

**DOI:** 10.1155/2014/203435

**Published:** 2014-01-23

**Authors:** Paula S. Ramos, Stephanie R. Shaftman, Ralph C. Ward, Carl D. Langefeld

**Affiliations:** ^1^Department of Medicine, Medical University of South Carolina, Charleston, SC 29425, USA; ^2^Department of Public Health Sciences, Medical University of South Carolina, Charleston, SC 29425, USA; ^3^Department of Public Health Sciences, Wake Forest School of Medicine and Center for Public Health Genomics, Winston-Salem, NC 27157, USA

## Abstract

The reasons for the ethnic disparities in the prevalence of systemic lupus erythematosus (SLE) and the relative high frequency of SLE risk alleles in the population are not fully understood. Population genetic factors such as natural selection alter allele frequencies over generations and may help explain the persistence of such common risk variants in the population and the differential risk of SLE. In order to better understand the genetic basis of SLE that might be due to natural selection, a total of 74 genomic regions with compelling evidence for association with SLE were tested for evidence of recent positive selection in the HapMap and HGDP populations, using population differentiation, allele frequency, and haplotype-based tests. Consistent signs of positive selection across different studies and statistical methods were observed at several SLE-associated loci, including *PTPN22*, *TNFSF4*, *TET3-DGUOK*, *TNIP1*, *UHRF1BP1*, *BLK*, and *ITGAM* genes. This study is the first to evaluate and report that several SLE-associated regions show signs of positive natural selection. These results provide corroborating evidence in support of recent positive selection as one mechanism underlying the elevated population frequency of SLE risk loci and supports future research that integrates signals of natural selection to help identify functional SLE risk alleles.

## 1. Introduction

Systemic lupus erythematosus (SLE) is an autoimmune disease whose prevalence, incidence, and disease severity are known to vary among ethnic groups. Increased prevalence has been reported among African-Americans, Asians, Hispanics, and Native Americans (reviewed elsewhere [1, 2]). The reasons for the ethnic disparities remain elusive. According to the “hygiene hypothesis” first proposed by Strachan two decades ago [3], the increased disease prevalence of autoimmune and allergic diseases in industrialized countries may be due to modern society's limited pathogen exposure. The Hygiene Hypothesis posits that humans have adapted to infectious exposures that were the norm in the past and that exposure was protective against autoimmune disease. Over many generations environmental pressure may have favored alleles that allow humans to respond to immune system challenges differently but resulted in an increased risk of autoimmune diseases. This could be a mechanism explaining the number of SLE risk alleles that are common in the population.

Human genome variation at the population level is shaped by four evolutionary processes: mutation, migration, random genetic drift, and natural selection. *Natural selection* is the process by which a trait, in the context of the organism's environment, becomes either more or less common in a population as a function of the effect of the inherited trait on the differential reproductive success. This ability to survive and reproduce and contribute to the gene pool of the next generation is known as *fitness*. Natural selection drives *adaptation*, the evolutionary process whereby over generations the members of a population become better suited to survive and reproduce in that environment. While *negative selection* decreases the prevalence of traits that diminish individuals' fitness, *positive selection* increases the prevalence of adaptive traits. Left untreated, SLE would have a reproductive fitness cost, defined as the ability to raise offspring that successfully reproduce. Thus, some evolutionary process must sustain the relative high frequency of SLE risk alleles seen in current populations around the world. We hypothesize that since the human genome is shaped by adaptation to environmental pressures at the population level, one plausible reason for the higher frequency of disease-risk alleles may be the direct effect of population-specific positive natural selection.

There is compelling evidence that natural selection is acting on a significant fraction of all genes (~3%) [4–7] and as much as 10% of the human genome [8]. Multiple studies have identified genes involved in immune-related functions to be under selection [8–10], including the *HLA* [11–14] (associated with all autoimmune diseases), *BTLA* [10] (associated with rheumatoid arthritis), *ITPR3* [10] (SLE, type 1 diabetes, Grave's disease), *PTPN22* [10] (rheumatoid arthritis, Crohn's disease, type 1 diabetes, vitiligo), *ITGAX* [10] (SLE), and *BLK* [10] (SLE, rheumatoid arthritis, Kawasaki disease). Finally, we have recently provided evidence that variants within the *APOL1* gene known to be under selective pressure in some African populations predispose to end-stage kidney disease in SLE [15]. Given the increasing evidence of selection at loci associated with human autoimmune diseases, identification of alleles under selection may provide further insight into SLE susceptibility and help understand the natural history of SLE predisposition.

## 2. Methods

A list of genetic regions with compelling evidence of association with SLE was compiled from the literature. This list includes results that met genome-wide significance in any genome-wide association study (GWAS) or transethnic study of SLE and common or rare variants that are considered established SLE-predisposing loci from candidate gene and other studies. The list of regions was based on the literature as of August 2013 and comprises 89 genes in 74 genomic regions.

This list was built upon all the SLE-associated regions described in recent reviews [16–19], which include common and rare variants from candidate gene studies with compelling evidence of association with SLE. We included all reported risk variants for SLE using data from the National Human Genome Research Institute's Catalog of Published GWAS (http://www.genome.gov/gwastudies) accessed on August 30th, 2013 [20]. Finally, we searched PubMed (http://www.ncbi.nlm.nih.gov/pubmed) for all large-scale transethnic or multiracial studies in SLE and catalogued all variants with a reported meta-analysis *P*  value < 5 × 10^−7^. The references for these more recent studies are included in Table 1. Given the paucity of studies conducted in some minority populations, and in order to avoid differential bias due to the number of reported associations in different ethnic groups, we chose to include all variation regardless of the population(s) where they were reported and ignore the information about the population(s) where they have been reported to date.

Assuming no other influencing factors, the advantageous alleles at a locus under positive selective pressure will tend to stochastically increase in prevalence over generations. This can lead to allele frequency differences between populations, which can be detected using statistics that compare the genetic variability within and between populations [69]. It can also lead to the haplotype carrying the advantageous allele to remain longer than genetic distance predicts around alleles of equal frequency, which can be measured using haplotype-based statistics [7]. The evidence of selection in each SLE-associated region was analyzed using both population differentiation, allele frequency spectrum, and haplotype-based statistics in the HapMap II and HGDP populations as implemented in the Haplotter (http://haplotter.uchicago.edu/) [7] and the Human Genome Diversity Project (HGDP) Selection Browsers (http://hgdp.uchicago.edu/cgi-bin/gbrowse/HGDP/) [70], respectively.

Haplotter displays the results of a scan for positive selection in the human genome using the International HapMap Project data (http://haplotter.uchicago.edu/) [7]. These data consist of ~800,000 polymorphic SNPs in three distinct population samples of unrelated individuals: 89 Japanese and Han Chinese individuals from Tokyo and Beijing, respectively, denoted as East Asian (ASN), 60 individuals of northern and western European origin (CEU), and 60 Yoruba (YRI) from Ibadan, Nigeria. It shows results on the autosomes only. Results from several selection statistics are displayed, including (1) the fixation index (*F*_ST_), (2) the Tajima's *D*, and (3) the integrated haplotype score (iHS). In situations where selection is restricted to certain populations or geographical locations, the allele frequencies at the locus that is undergoing selection may vary significantly between different populations. The fixation index *F*_ST_ provides a metric of the magnitude of global allele frequency differentiation between populations at a locus [69, 71]. *F*_ST_ is directly related to the variance in allele frequency among populations and, conversely, to the degree of resemblance among individuals within populations. If *F*_ST_ is small, it means that the allele frequencies within each population are similar; if it is large, it means that the allele frequencies are different [72]. The Tajima's *D* is based on the frequencies of the polymorphisms segregating in a locus [73]. As described [7], positive selection results in an excess of high frequency derived alleles compared to neutral expectations when the selected allele has swept to high frequencies. Positive selection also results in an excess of low frequency polymorphisms, especially when the selected allele is close to fixation or right after fixation. This skewing of SNP frequencies in different directions can be detected by Tajima's *D*, which is based on the frequencies of SNPs segregating in the region of interest [73]. Signals of selective sweeps will result in high negative *D*. The integrated haplotype score (iHS) uses the lengths of the haplotypes surrounding each core SNP to identify SNPs for which alleles have rapidly risen in frequency [7, 74]. It is based on linkage disequilibrium (LD) surrounding a positively selected allele compared with background, providing evidence of recent positive selection at a locus [7]. An iHS score > 2.0 reflects the fact that haplotypes on the ancestral background are longer compared with those on the derived allelic background.

For these analyses, genome-wide SNP data from Phase II of the HapMap Project were used to investigate if the regions associated with SLE showed evidence of selection in the CEU, YRI, and ASN populations using these three metrics (iHS, Tajima's *D*, and *F*_ST_). Regions of 1 Mb around each of the 74 regions in Table 1 were queried, and, when higher than 2, the maximum value on the *Y*-axis (−log⁡(*Q*)) in this 1 Mb interval was recorded. As described by Voight et al. [7], the −log⁡(*Q*) value represents the negative log of the rank of the observed statistic for a given SNP divided by the total number of SNPs. The statistic that is ranked is obtained independently for each of the three statistics separately for each population. For *D*, the estimated value of *D* was used for ranking. For iHS, for each SNP, 25 SNPs on either side of the SNP are scanned for |iHS| > 2. The proportion of SNPs in this 51 SNP window with |iHS| > 2 is computed. For *F*_ST_, the statistic to be ranked is obtained in a similar manner as that for iHS except for each population comparison, the thresholds for defining a significant *F*_ST_ is based on the top 5% cutoff for each population comparison. The different thresholds used for *F*_ST_ were CEU-YRI: 0.2976, CEU-ASN: 0.2055, and YRI-ASN: 0.3374. Haplotter also displays the *F*_ST_ value of the SNPs in the top 1% within each population comparison, which were also recorded, if any such SNPs were present in the 1 Mb interval. In addition to these, Haplotter shows an empirical *P* value estimated for each gene and for each population, as detailed by Voight et al. [7]. When this *P* value showed significant evidence for selection, the value was recorded.

The HGDP Selection Browser displays results from a series of genome-wide scans for natural selection using single nucleotide polymorphism (SNP) genotype data from the Human Genome Diversity-CEPH Panel (HGDP), a dataset containing 938 individuals from 53 populations typed on the Illumina 650Y platform (http://hgdp.uchicago.edu/cgi-bin/gbrowse/HGDP/) [70]. Summary statistics regarding haplotype structure and population differentiation on this data can be queried in the browser. These include the iHS, the *F*_ST_, and the cross-population extended haplotype homozygosity test (XP-EHH) [74]. While the iHS detects partial selective sweeps of moderate frequency (~50%–80%), the XP-EHH detects selected alleles that have risen to near fixation in one population (above 80% frequency) [7, 74]. As described by Pickrell et al. [70], the *F*_ST_ was calculated on the level of population groupings identified by Rosenberg et al. [75]; that is, if a SNP has high *F*_ST_, most of the variance in allele frequencies is captured by the seven labels identified in that paper. In the browser, plotted is the −log⁡_10_ of the empirical *P* value for each SNP—the higher this plotted  −log⁡_10_ *P* value, the more extreme (high) the *F*_ST_ value is compared the rest of the genotyped SNPs. The iHS was calculated as in Voight et al. [7] and smoothed across windows. Plotted is the −log⁡_10_ of the *P* value for a window centered at the SNP; high values again indicate potential signals of positive selection. The test statistic was the fraction of SNPs with |iHS| > 2. The XP-EHH was calculated as in Sabeti et al.'s work [74]. The test statistic was the maximum XP-EHH. Again, the plotted measure is a measure of how extreme a SNP is with regard to the rest of the genome, and high values indicate outliers potentially due to the action of natural selection. The iHS and XP-EHH have been calculated in each individual population, as well as in the following groupings: Bantu-speaking populations, Europeans, Middle Easterners, Central Asians, East Asians, Americans, and Oceanians.

Regions of 1 Mb around each of the 74 regions in Table 1 were queried, and the maximum value on the *Y*-axis (−log⁡(*P*)) in this 1 Mb interval was recorded.

## 3. Results

To test whether SLE susceptibility loci show evidence of positive selection, a list of 74 genetic regions with compelling evidence of association with SLE was compiled (Table 1). In order to test whether SLE-associated loci show evidence for recent positive selection, 1 Mb regions around each of the 74 regions were queried. Regions where the maximum −log⁡(*Q*) > 3 (for Haplotter) or −log⁡(*P*) > 3 (for HGDP) for the *F*_ST_, *D*, iHS, or XP-EHH were considered as showing evidence for recent positive selection (Tables 2 and 3). In addition, regions that in the HapMap populations had SNPs with *F*_ST_ values in the top 1% within each population comparison, or whose empirical *P* value estimated for each gene and for each population showed significant evidence for selection (*P* value < 0.001) were also considered to show evidence for selection. Of the 74 regions associated with SLE, 19 showed evidence of selection in a HapMap population (Table 2), and 16 exhibited a signal of selection in a HGDP population (Table 3). Many of these loci also had corroborating evidence using different metrics.

In the HapMap data multiple regions displayed evidence of population differentiation, as indicated by the *F*_ST_, which was the highest in the *PTPN22*, *TET3-DGUOK*, *ITPR3*, *ITGAM*, and *CD226* regions. Several SNPs with very high *F*_ST_ (in the top 1% within each population comparison) were identified in these and other regions, especially *XKR6-BLK* (*F*_ST_ = 0.92 in YRI versus ASN), *TET3-DGUOK* (*F*_ST_ = 0.85 in YRI versus ASN, and *F*_ST_ = 0.80 in YRI versus CEU), *CD226* (*F*_ST_ = 0.80 in CEU versus YRI), *LRRC18-WDFY4* (*F*_ST_ = 0.80 in YRI versus ASN), *IFIH1* (*F*_ST_ = 0.78 in CEU versus YRI), *PTPN22* (*F*_ST_ = 0.75 in YRI versus ASN), and *ITGAM* (*F*_ST_ = 0.75 in YRI versus ASN). The highest allele frequency differences, as indicated by the *D* statistic, were detected in the *PTPN22*, *IFIH1*, *ITPR3*, and *XKR6-BLK* regions. The *ITPR3* region also had a high iHS. This and *BLK* are the regions that displayed the most consistently strong evidence for selection according to all three metrics. The *ITPR3* gene lies at 6p21, adjacent to the centromeric end of the extended MHC region, after the class II flanking region. *XKR6* and *BLK* lie on the same chromosomal inversion at 8p23.1. *PTPN22*, *ITPR3*, and *CD226* exhibited the strongest evidence for selection according to the frequency-based statistics. Finally, several regions included genes whose empirical *P* value showed significant evidence for selection. These genes included *XKR6* (*P* = 0.004 in ASN) and *UHRF1BP1* (*P* = 0.006 in CEU). Other genes were significant in several regions, such as the *TET3-DGUOK* region (*DUSP11* and *STAMBP* with *P* = 0.005 and *P* = 0.007, resp., in CEU). The *PTPN22*, *ITGAX* (near *ITGAM*), *ITPR3*, and *BLK* regions were recently reported to be under selection (in YRI, YRI, YRI, and ASN, resp.) in a candidate gene study by Grossman et al. [10], who used full-genome sequence variation from the 1000 Genomes Project and the composite of multiple signals (CMS) test.

Since the regions in Table 2 showed evidence of selection in the HapMap samples, the evidence centered at the specific SNP associated with SLE were tested (Supplementary Table 1 in the Supplementary Material available online at http://dx.doi.org/10.1155/2014/203435). Specifically, Haplotter displays the iHS and *F*_ST_ for common SNPs. Of the queried SLE-associated SNPs, the highest evidence of population differentiation was shown by rs9937837 in *ITGAM* (*F*_ST_ = 0.81 in YRI versus ASN). Evidence for association according to the iHS test was observed in *CFHR1-CFHR4* (rs16840639, iHS = −2.63 in YRI), *NMNAT2* (rs2022013, iHS = 2.50 in ASN), *APOBEC4* (rs10911390, iHS = −2.36 in ASN), *CFH* (rs6677604, iHS = −2.30 in YRI), *UHRF1BP1* (rs11755393, iHS = −2.28 in CEU), and *CD226* (rs727088, iHS = 2.14 in CEU). The evidence for selection at the *UHRF1BP1* variant was recently reported in a study of candidate inflammatory-disease SNPs using the same statistic and HapMap II data [76].

**Figure 1:**
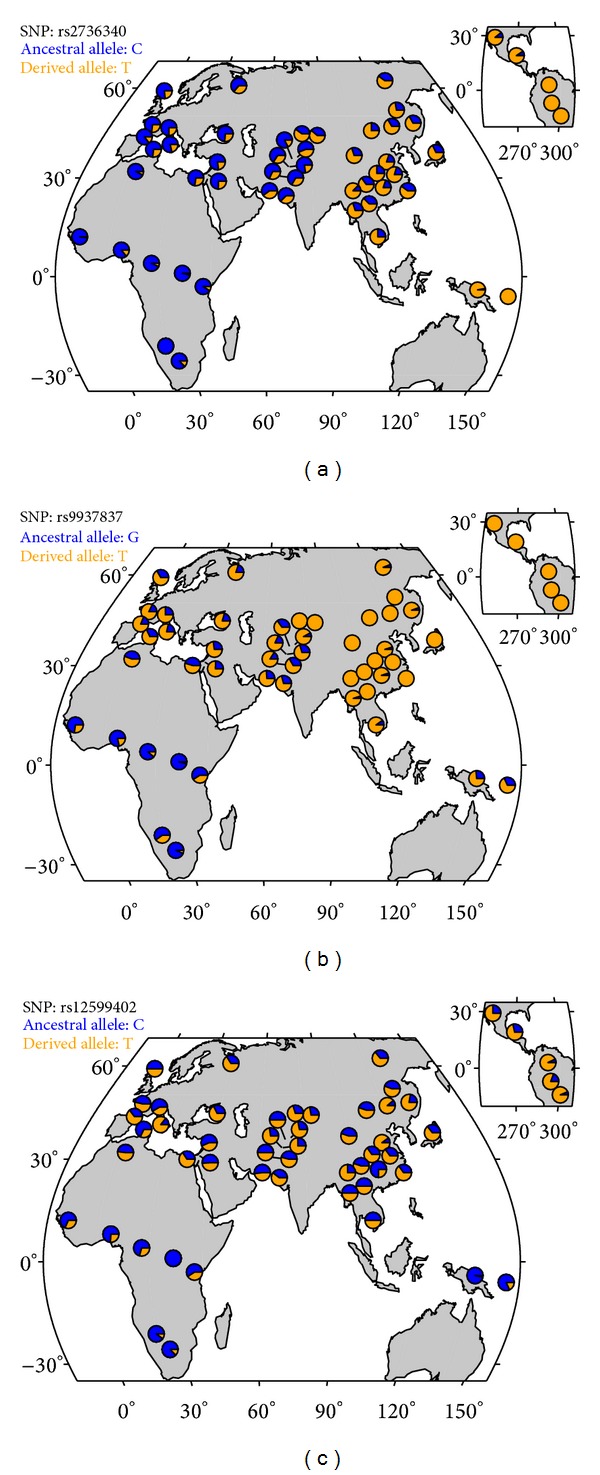
Worldwide distribution of allele frequencies for SLE-associated SNPs rs2736340 in *BLK* (a), rs9937837 in *ITGAM* (b), and rs12599402 in *CLEC16A* (c).

In the HGDP data, the highest XP-EHH was detected in the *BLK*, *CLEC16A*, and *IRF8 *regions and the maximum iHS in the *CLEC16A* and *PTTG1* regions. The *CLEC16A*, *BLK*, *PTPN22*, and *UHRF1BP1* regions showed strong evidence for selection under the haplotype-based statistics. *TNFSF4*, *IL10*, and *BLK* were the regions showing the highest degree of population differentiation. The *TNFSF4* and *BLK* regions showed the strongest most consistent evidence of selection according to all three metrics. Using the same HapMap II data, Raj and colleagues [76] previously reported SNPs with a significant signal of selection in *CLEC16A* (rs12708716, iHS = 2.29 in CEU) and *UHRF1BP1* (rs11755393, iHS = −2.28 in CEU). As mentioned, the *BLK* and *ITGAX-ITGAM* regions were recently reported to be under selection (in ASN and YRI, resp.) in a candidate genes study using the 1000 Genomes Project samples [10]. For the genes in Table 2, an inspection of the worldwide distribution of allele frequencies for the SNPs associated with SLE (Supplementary Table 2) revealed interesting patterns for SNPs in *BLK*, *ITGAM*, and *CLEC16A* (Figure 1).

Comparing the results of the tests for selection in the HapMap and the HGDP samples shows that there are seven genetic regions captured by at least one test in both datasets (Table 4). The common regions captured by the majority of tests were that of the *PTPN22*, *UHRF1BP1*, and *BLK* genes. While the region of the *TNIP1* gene was captured in both the HapMap and HGDP populations by the frequency spectrum and population differentiation statistics (*D* and *F*_ST_), the region of the *UHRF1BP1* gene was captured by the haplotype-based statistics. The evidence for selection in these seven genetic regions (Table 4) is strengthened by the fact that they show consistent evidence across different studies and analytic methods.

## 4. Discussion

The diversity exhibited in the human genome is a result of stochastic population genetics processes such as mutation, migration, drift, and selection. SLE disproportionately affects women of child bearing age and without treatment would tend to put affected individuals at a reproductive disadvantage; here, reproductive disadvantage not only includes conception but the ability to raise offspring that successfully reproduce. Thus, strong alternative forces or changing selective pressure must exist that permits the relative high frequency of these risk alleles seen in current populations around the world. Infectious diseases and pathogenic exposures have been postulated to be important factors resulting in strong selective pressure and might provide such alternative pressures. This study investigated whether SLE susceptibility loci show signs of recent positive selection by comparing these regions to the background distribution of genetic variation.

Two important studies have computed several genome-wide tests for selection in two main reference populations, the HapMap and the HGDP populations [7, 70], and implemented the results in genetic browsers. These browsers were queried to assess whether SLE-associated genetic regions have shown evidence for selection in the HapMap and HGDP populations.

This study reports several SLE-associated loci that show evidence for selection in the HapMap populations, and several SLE-associated loci that show evidence for selection in the HGDP populations. Seven genetic regions showed evidence for selection on both the HapMap and HGDP populations. These include the regions of the *PTPN22*, *TNFSF4*, *TET3-DGUOK*, *TNIP1*, *UHRF1BP1*, *BLK*, and *ITGAM* genes. In addition to the regions that are concordant, the different results obtained with the different metrics and datasets are expected, mostly due to the different coverage of the SNP arrays used, local adaptation in different ethnic groups, and the different test statistics which are likely recovering selective events from different time periods and for different stages of the selective sweep [77].

Several of these genes have been previously reported to show patterns of genetic variation that are consistent with evidence for recent positive selection. For example, in their search for inflammatory-disease SNPs that localize to regions of the genome where patterns of genetic variation are consistent with that expected under a model of recent positive selection, Raj and colleagues [76] also reported SNPs in *CLEC16A* and *UHRF1BP1* that exhibit a significant signal of selection using the iHS test. Furthermore, they show that the SLE susceptibility allele in *UHRF1BP1* is associated with decreased *UHRF1BP1* RNA expression in different cell subsets, suggesting that the SLE risk allele is under recent selection and has a regulatory effect [76]. Furthermore, *UHRF1BP1* has been shown to be significantly differentially expressed in dendritic cells after Mycobacterium tuberculosis (MTB) infection [78]. Using full-genome sequence variation from the 1000 Genomes Project and the composite of multiple signals (CMS) test, Grossman et al. [10] reported the *PTPN22*, *ITGAX* (near *ITGAM*), *ITPR3*, and *BLK* regions to show evidence for recent positive selection.

Several of the immune genes that have been identified in regions under selection are under the selective pressure of known pathogens, such as the Duffy blood group atypical chemokine receptor (*DARC*) gene to *Plasmodium vivax* malaria [79], ras homolog family member A (*RHOA*), and OTU domain ubiquitin aldehyde binding 1 (*OTUB1*) genes to *Yersinia pestis* (plague) [80], or the tyrosylprotein sulfotransferase 1 (*TPST1*) gene to *HIV* [81]. Several genetic regions associated with susceptibility to different autoimmune diseases show evidence of selection that has been attributed to host-pathogen coevolution, including the multiple major histocompatibility complex (MHC) [82–84] and the celiac risk locus *SH2B3* as a protective factor against bacterial infection [85]. Karlsson et al. [86] have recently reported that cholera has exerted strong selective pressure on proinflammatory pathways, and Jostins et al. [87] reported considerable overlap between susceptibility loci for inflammatory bowel disease and mycobacterial infection. Variants in the *IFIH1* gene, whose protein is a cytoplasmic helicase that recognizes RNA of picornaviruses and mediates induction of interferon response to viral RNA, have been shown to affect *IFIH1* function and host antiviral response [88]. In the context of SLE predisposing loci, Clatworthy et al. [89] have shown that *FCGR2B* is important in controlling the immune response to *Plasmodium falciparum*, the parasite responsible for the most severe form of malaria, and suggests that the higher frequency of human *FCGR2B* polymorphisms predisposing to SLE in Asians and Africans may be maintained because these variants reduce susceptibility to malaria. The complement component (3b/4b) receptor 1 (*CR1*) gene has been shown to be a *P. falciparum* resistance gene [90] used by the parasite for host invasion. Machado et al. [91] have suggested that helminth infection has driven positive selection of *FCGR*s variation. Finally, Grossman et al. [10] implicated *Salmonella typhimurium* and other exposures that directionally drive selection of the toll-like receptor 5 (*TLR5*) gene [92]. Given that infectious organisms are strong agents of natural selection, it is plausible that alleles selected for protection against infection predispose to autoimmune diseases.

It is important to acknowledge the challenges and limitations inherent to the study of traits with complex genetic architectures and/or a less clear influence on survival and reproduction, such as SLE. As Castiblanco and colleagues [93] recently articulated, the differences in allele and genotype frequencies of diverse human populations depend upon their evolutionary and epidemiological history, including environmental exposures, which might explain why some risk alleles to autoimmunity may be protective factors to infectious diseases and vice versa in a given population (e.g., *PTPN22* [94, 95] and TNF [96]). Immune and infectious agents have been recognized as among the strongest selective pressures for natural populations, as shown by the identification of candidate adaptive alleles that functionally contribute to biological variation in contemporary populations. However, clarifying the relationship between the functional alleles and reproductive fitness in the environment in which they rose to a high frequency in the ancestors of the study population can rarely be attained. In complex diseases such as SLE, despite the established associations to specific regions or polymorphisms, the true causal variants still remain largely unknown. The emerging availability of genome-wide functional data allows the integration of an unprecedented amount of biological information to help identify potential functional variants and characterize their biological impact. Recent examples demonstrate how the integration of signatures of positive selection with phenotypic association studies and/or with regulatory data can improve the identification of functional loci [10, 97–99]. Also, the complex genetic architecture of SLE, resulting from the effects of many alleles of small effects, suggests that adaptation is likely to have occurred by simultaneous selection on variants at many loci. In this scenario, the response to selection is due to small frequency shifts of many alleles. However, most methods to detect selection rely on rapid fixation of strongly selected alleles. The development of novel analytical approaches to detect more subtle signatures of selection will improve the identification of selection signatures in complex diseases like SLE. Clearly, much remains to be done until the functional adaptive SLE risk loci are identified, the phenotypic consequences of these risk alleles elucidated, and the relationship between the functional alleles and reproductive fitness clarified. Recent progresses will provide the necessary tools to accelerate the discovery of these functional adaptive variants that increase the risk of SLE, which will improve knowledge about the etiology and deepen our understanding of the natural history of SLE. Further research regarding exploration of the interplay between infection, type of exposure, additional environmental factors, and autoimmunity will result in the discovery of multiple factors underpinning perhaps newly identified physiopathology mechanisms of SLE and autoimmune diseases [93].

In summary, this study has systematically queried the HapMap and HGDP populations for evidence for selection at SLE susceptibility regions and provides a comprehensive catalog of regions with both evidence for recent positive selection and association with SLE. These results provide support for recent positive selection influencing genetic variation associated with SLE, suggesting that population-specific selective pressures may be one of the factors behind the high frequency of SLE risk alleles in the population and differential disease risk. Finally, these results support future analyses aimed at identifying the specific selective pressures and characterizing the functional mechanisms of adaptation and disease predisposition.

## Supplementary Material

Supplementary Material shows the specific SLE-associated SNPs queried for evidence for selection in the HapMap and HGDP samples.

## Figures and Tables

**Table 1:** Genetic regions with compelling evidence for association with SLE.

Gene(s) region	Chr	Pos (Mb)
C1q [21]	1	22.96
IL12RB2 [22]	1	67.55
PTPN22 [22–25]	1	114.16
FCGR2A, FCGR3A [26, 27]	1	159.74
TNFSF4 [22, 28–33]	1	171.42
NMNAT2 [22, 24, 32]	1	181.48
NCF2 [22]	1	181.79
APOBEC4 [28]	1	181.88
CFH [34]	1	194.89
CFHR1, CFHR4 [34]	1	196.79
CRP [35]	1	199.72
IL10 [22]	1	205.01
LYST [22]	1	233.89
RASGRP3 [28, 32]	2	33.51
TET3, DGUOK [28]	2	74.21
IFIH1 [22, 36]	2	162.83
STAT4 [22–24, 32, 37–41]	2	191.60
PDCD1 [42]	2	242.44
SCN10A [28]	3	38.71
TREX1 [43]	3	48.48
DNASE1L3 [44]	3	58.15
PXK [22–24]	3	58.29
TMEM39A [45]	3	120.63
CD80 [28]	3	120.73
AFF1 [46]	4	88.15
BANK1 [23, 28, 47]	4	102.93
LEF1 [46]	4	109.19
IL21 [48]	4	123.75
PPP2CA [49]	5	133.53
TNIP1 [22, 28, 32]	5	150.39
PTTG1 [22, 32]	5	159.78
C4 [50]	6	32.09
HLA-DRB1 [24, 39, 51–53]	6	32.59
ITPR3 [54]	6	33.70
UHRF1BP1 [22, 28]	6	34.87
BACH2 [28]	6	90.69
ATG5, PRDM1 [22–24, 28]	6	106.53
TNFAIP3 [22, 28, 32, 38, 55]	6	138.23
ICA1 [22, 24]	7	8.12
JAZF1 [22]	7	27.84
IKZF1 [28, 32]	7	50.31
IRF5, TNPO3 [22, 24, 28, 39, 40, 56, 57]	7	128.37
XKR6 [24]	8	10.79
BLK [22–24, 28, 39, 40]	8	11.39
LYN [24]	8	56.95
ARMC3 [22]	10	23.26
LRRC18, WDFY4 [28, 32, 33]	10	49.89
ARID5B, RTKN2 [28]	10	63.94
SLC29A3 [28]	10	72.75
PHRF1, IRF7 [22–24]	11	0.58
CD44, PDHX [28, 58]	11	34.94
DDX6 [22, 32]	11	118.13
ETS1 [28, 32, 33]	11	127.83
CREBL2, GPR19, CDKN1B [28]	12	12.66
DRAM1 [28]	12	102.27
SLC15A4 [28, 32]	12	127.84
ELF1 [28]	13	40.40
C2 [59]	14	20.75
CSK [60]	15	72.86
DNASE1 [61]	16	3.64
CLEC16A [28]	16	11.04
PRKCB [62]	16	23.75
SEZ6L2 [28]	16	29.79
ITGAM, ITGAX [23, 24, 28, 63]	16	31.18
IRF8 [22, 45]	16	84.49
IKZF3, ZPBP2 [45]	17	37.91
CD226 [22, 57, 64]	18	65.68
TYK2 [22, 57]	19	10.32
ICAM1, ICAM4, ICAM5 [65]	19	10.40
ACP5 [66]	19	11.55
DDA1 [28]	19	17.28
UBE2L3 [22–24, 28]	22	20.25
SCUBE1 [24]	22	41.93
IRAK1, MECP2 [22, 23, 67, 68]	X	152.93

The reference list for each gene region does not intent to be exhaustive; instead, only the first and/or strongest associations reported to date are mentioned. A comprehensive list of all the studies that report each region have been recently reviewed elsewhere [16–18]. Chr: chromosome; Pos: position (in Mega basepairs) according to Human Genome Build hg18.

**Table 2:** Regions with evidence for selection on the HapMap populations.

Gene region	Chr	Mb	iHS		*D*		F_ST_			Empirical *P* value	
			Max −log⁡(*Q*)	Pop	Max −log⁡(*Q*)	Pop	Max −log⁡(*Q*)	Value	Pop	Min *P* value	Pop
PTPN22	1	114.158	—	—	3.6	YRI	3.2	0.75	YRI versus ASN	—	—
TNFSF4	1	171.419	2.5	ASN	2.3	ASN	2.7	0.60	YRI versus ASN	0.005	ASN
NMNAT2	1	181.484	2.5	ASN	2.4	CEU	—	—	—	0.004	ASN
NCF2	1	181.791	2.5	ASN	—	—	—	0.65	CEU versus YRI	0.004	ASN
APOBEC4	1	181.882	2.5	ASN	—	—	—	0.65	CEU versus YRI	0.004	ASN
CFH	1	194.888	—	—	—	—	3.0	0.60	YRI versus ASN	—	—
CFHR1, CFHR4	1	196.789	2.0	YRI	—	—	3.0	0.60	YRI versus ASN	—	—
TET3, DGUOK	2	74.212	2.7	CEU	2.6	ASN	3.2	0.85	YRI versus ASN	0.001	CEU
IFIH1	2	162.832	—	—	3.8	CEU	2.2	0.78	CEU versus YRI	—	—
TREX1	3	48.481	2.4	ASN	2.1	ASN	—	—	—	0.002	ASN
TNIP1	5	150.390	—	—	3.0	CEU	—	0.65	CEU versus YRI	—	—
ITPR3	6	33.697	3.4	YRI	3.3	YRI	3.3	0.60	YRI versus ASN	—	—
UHRF1BP1	6	34.868	2.5	CEU	2.4	YRI	—	0.50	—	0.004	CEU
XKR6	8	10.791	2.7	ASN	3.3	ASN	2.6	0.92	YRI versus ASN	0.003	ASN
BLK	8	11.389	2.7	ASN	3.2	ASN	2.6	0.92	YRI versus ASN	0.005	ASN
ARMC3	10	23.257	—	—	2.5	CEU	2.5	0.65	YRI versus ASN	—	—
LRRC18, WDFY4	10	49.893	—	—	2.0	ASN	2.5	0.80	YRI versus ASN	—	—
ITGAM	16	31.179	—	—	—	—	3.4	0.75	YRI versus ASN	—	—
CD226	18	65.681	—	—	3.1	CEU	3.7	0.80	CEU versus YRI	—	—

Regions were considered to show evidence for selection if the maximum −log⁡(*Q*) > 3 for either the *F*_ST_, *D*, or iHS, or it had SNPs with F_ST_ values in the top 1% within each population comparison, or the empirical *P* value estimated for the SLE-associated gene and for each population showed significant evidence for selection (*P* value < 0.01). Cells that did not meet these thresholds or whose −log⁡(*Q*) > 2 are marked with (—). The table shows the highest −log⁡(*Q*) value and respective population for the iHS, *D*, and F_ST_, the F_ST_ statistic (value) for SNPs in the top 1% and the population comparison, and the minimum empirical *P* value in each region. *Q* is the rank of the observed statistic for a given SNP divided by the total number of SNPs. The statistic that is ranked is obtained independently for each of the three statistics separately for each population. For iHS, for each SNP, 25 SNPs on either side of the SNP are scanned for |iHS| > 2. The proportion of SNPs in this 51 SNP window with |iHS| > 2 is computed. For *D*, the estimated value of *D* was used for ranking. For F_ST_, the statistic to be ranked is obtained in a similar manner as that for iHS except for each population comparison, the thresholds for defining a significant F_ST_ is based on the top 5% cutoff for each population comparison. See Methods for details. Chr: chromosome, Mb: mega basepairs, Max: maximum, Min: minimum, Pop: population, ASN: East Asian, CEU: European, YRI: African.

**Table 3:** Regions with evidence for selection in the HGDP populations.

Gene region	Chr	Mb	*F*_ST_	iHS		XP-EHH	
			Max −log⁡(*P*)	Max −log⁡(*P*)	Pop	Max −log⁡(*P*)	Pop
PTPN22	1	114.158	2.5	3	Afr	3.5	Afr
TNFSF4	1	171.419	4.5	2.5	EAsia	3.5	EAsia
CRP	1	199.719	3.5	—	—	2.5	Afr, Eur
IL10	1	205.008	4	2	MEast, EAsia	2.5	SAsia EAsia
TET3, DGUOK	2	74.212	2.5	2	SAsia	3.5	MEast, SAsia
TNIP1	5	150.390	3.5	1.5	MEast	3	Amer
PTTG1	5	159.781	—	3.5	Afr	2.8	MEast, Afr
UHRF1BP1	6	34.868	—	3	Amer	3.5	Amer
IKZF1	7	50.315	3.5	3	EAsia	2.5	EAsia
BLK	8	11.389	4	3	SAsia, MEast, Afr	4	EAsia
ARMC3	10	23.257	2.5	2.5	MEast	3.5	MEast
SLC15A4	12	127.844	3.5	—	—	2.5	Afr, Eur
CLEC16A	16	11.038	2	4	Amer	4	Amer
ITGAM	16	31.179	2.5	2	EAsia	3.5	EAsia
IRF8	16	84.490	2.5	2	SAsia	4	SAsia
SCUBE1	22	41.929	2.5	2	Oceania	3	Oceania

Regions were considered to show evidence for selection if the maximum −log⁡_10_(*P*) > 3 for either the *F*_ST_, iHS, or XP-EHH. The table shows the highest −log⁡_10_(empirical *P* value) and respective population for the *F*_ST_, iHS, and XP-EHH in each region. Regions whose −log⁡_10_(*P*) < 2 are marked with (—). See Methods for details. Chr: chromosome, Mb: mega basepairs, Max: maximum, Pop: population. Populations: Bantu-speaking Africans (Afr), Europeans (Eur), Middle Easterners (MEast), Eastern Asians (EAsia), South Asians (SAsia), Americans (Amer), and Oceanians (Oceania).

**Table 4:** Summary of regions with evidence for selection on both the HapMap and HGDP populations.

Gene region	HapMap					HGDP		
	iHS	*D*	*F*_ST_		Min empirical *P* value	*F*_ST_	iHS	XP-EHH
		Max −log⁡(*Q*)	Max −log⁡(*Q*)	Value		Max −log⁡(*P*)	Max −log⁡(*P*)	Max −log⁡(*P*)
PTPN22		3.6	3.2	0.75			3.0	3.5
TNFSF4				0.6	0.005	4.5		
TET3, DGUOK			3.2	0.85	0.001			3.5
TNIP1		3.0		0.65		3.5		3.0
UHRF1BP1	|2.28|*				0.004		3.0	3.5
BLK				0.92	0.005	4.0	3.0	4.0
ITGAM			3.4	0.75				3.5

Please refer to footnotes on Tables 2 and 3 for details. *iHS = −2.28 for rs11755393.
