# Supplementary material for: Genes Associated with SLE Are Targets of Recent Positive Selection
Source: Autoimmune Dis. 2014 Jan 23;2014:203435. doi: 10.1155/2014/203435 (PMC3920976; doi:10.1155/2014/203435)
Supplement: Supplementary file 1 — Supplementary Material shows the specific SLE-associated SNPs queried for evidence for selection in the HapMap and HGDP samples. [file 203435.f1.pdf]

Supplementary Material

Supplemental Table 1. SLE-associated SNPs queried for evidence for selection in the HapMap samples.

Region	SNPs
PTPN22	rs2476601
TNFSF4	rs2205960
TNFSF4	rs2205960
TNFSF4	rs3850641
NMNAT2	rs2022013
NCF2	rs10911363
APOBEC4	rs10911390
CFH	rs6677604
CFHR1,CFHR4	rs16840639
TET3,DGUOK	rs6705628
TET3,DGUOK	rs4852324
TET3,DGUOK	rs6705628
IFIH1	rs1990760
TREX1	rs72556554
TNIP1	rs7708392
TNIP1	rs10036748
ITPR3	rs3748079
UHRF1BP1	rs11755393
UHRF1BP1	rs3734266
XKR6	rs6985109
BLK	rs13277113
BLK	rs2736340
BLK	rs2254546
BLK	rs13277113
ARMC3	rs11013210
LRRC18,WDFY4	rs1913517
LRRC18,WDFY4	rs877819
ITGAM	rs1143679
ITGAM	rs9888739
ITGAM	rs4548893
ITGAM	rs9937837
CD226	rs763361
CD226	rs727088

Supplemental Table 2. SLE-associated SNPs queried for evidence for selection in the HGDP samples.

Gene region	SNPs
PTPN22	rs2476601
TNFSF4	rs2205960
TNFSF4	rs2205960
TNFSF4	rs3850641
CRP	rs3093061
IL10	rs3024505
TET3,DGUOK	rs6705628
TET3,DGUOK	rs4852324
TNIP1	rs7708392
TNIP1	rs10036748
PTTG1	rs2431099
UHRF1BP1	rs11755393
UHRF1BP1	rs3734266
IKZF1	rs4917014
IKZF1	rs10276619
BLK	rs13277113
BLK	rs2736340
BLK	rs2254546
BLK	rs13277113
ARMC3	rs11013210
SLC15A4	rs10847697
SLC15A4	rs1385374
CLEC16A	rs12599402
CLEC16A	rs12708716
ITGAM	rs1143679
ITGAM	rs9888739
ITGAM	rs4548893
ITGAM	rs9937837
IRF8	rs12444486
IRF8	rs11644034
SCUBE1	rs2071725
